# Reference interval of thyroid stimulating hormone and free thyroxine in a reference population over 60 years old and in very old subjects (over 80 years): comparison to young subjects

**DOI:** 10.1186/1756-6614-6-13

**Published:** 2013-12-24

**Authors:** Rosita Fontes, Claudia Regina Coeli, Fernanda Aguiar, Mario Vaisman

**Affiliations:** 1 Hospital Clementino Fraga Filho, Universidade Federal do Rio de Janeiro, Rua Prof. Rodolpho Paulo Rocco 255, Cidade Universitária, CEP 21941-913, Rio de Janeiro-RJ, Brazil; 2 Diagnósticos da América SA, Rio de Janeiro, Brazil; 3 Núcleo de Estudos de Saúde Coletiva, Universidade Federal do Rio de Janeiro, Rio de Janeiro, Brazil

## Abstract

**Background:**

Studies based on laboratory data about thyroid stimulating hormone (TSH) and free thyroxine (FT4) reference interval (RI) show conflicting results regarding the importance of using specific values by age groups with advancing age. Retrospective laboratory data or non-specific criteria in the selection of subjects to be studied may be factors leading to no clear conclusions. The aim of this study is to test the hypothesis that TSH and FT4 have specific RI for subjects over 60 to 80 years.

**Methods:**

We evaluated prospectively 1200 subjects of both sexes stratified by age groups, initially submitted to a questionnaire to do the first selection to exclude those with factors that could interfere in TSH or FT4 levels. Then, we excluded those subjects with goiter or other abnormalities on physical examination, positive thyroid peroxidase antibodies (TPOAb), thyroglobulin antibodies (TGAb), and other laboratory abnormalities.

**Results:**

TSH increased with age in the whole group. There was no statistical difference in the analysis of these independent subgroups: 20–49 *versus* 50–59 years old (p > 0.05), and 60–69 *versus* 70–79 years old (p > 0.05). Consequently, we achieved different TSH RI for the three major age groups, 20 to 59 years old: 0.4 - 4.3 mU/L, 60 to 79 years old: 0.4 - 5.8 mU/L and 80 years or more: 0.4 - 6.7 mU/L. Conversely, FT4 progressively decreases = significantly with age, but the independent comparison test between the sub-groups showed that after age 60 the same RI was obtained (0.7 - 1.7 ng/dL) although the minimum value was smaller than that defined by manufacturer. In the comparison between TSH data obtained by this study and those defined by the manufacturer (without segmentation by age) 6.5% of subjects between 60 and 79 years and 12.5% with 80 years or more would have a misdiagnosis of elevated TSH.

**Conclusions:**

TSH normal reference range increases with age, justifying the use of different RI in subjects 60 years old and over, while FT4 decreases with age. Using specific-age RI, a significant percentage of elderly will not be misdiagnosed as having subclinical hipothyroidism.

## Introduction

In recent decades there has been increased life expectancy of the population and, consequently, of the aging process. Persons older than age 60 comprise 20 per cent of the world population in the more developed regions, and from 5 to 8 per cent in the less developed regions. The oldest old, persons aged 80 years or older, is the fastest growing segment of the older population and by 2050 the number of this group is projected to be five times as large as at present [1].

Several aspects of the aging process affect the endocrine system and stimulate the use of screening programs for the detection of hormonal changes and drug interventions with hormone replacement therapies to provide better quality of life for the elderly. Evaluation of thyroid function in normal elderly is difficult, since the prevalence of non-thyroid disease and the use of medications that interfere with thyroid function is greater than in young people. As a result, questions about the meaning of functional changes observed in the elderly are relatively common [2].

Data interpretation of thyroid function in the elderly has been changing over the past decades. In a study conducted in 1995 in a non-selected population, the authors considered that subjects of any age with some degree of TSH elevation had some grade of thyroid gland failure [3]. However, in 2002, the NHANES III study revisited this parameter data in a population excluding those with evidence of thyroid disease and, in this more uniform population, TSH still showed a progressive increase with age [4].

The International Federation of Clinical Chemistry and Laboratory Medicine (IFCC) Committee developed the theory of reference values for the “Reference Intervals and Decision Limits (CRIDL)” [5]. In 1995 the Clinical and Laboratory Standards Institute (CLSI) first published with IFCC the joint guideline “Defining, Establishing, and Verifying Reference Intervals in the Clinical Laboratory”, reviewed in 2008 [6]. This document recommends application of prospective questionnaires and, if necessary, physical evaluation, of candidate subjects to be part of a control group. It also discourages the indirect approach in which database results are used to establish ranges, retroactively identifying acceptable reference populations. This has been a challenge since then, and many clinical laboratories do not have these procedures performed in accordance with the recommendations, due to the fact that they require time, additional costs, knowledge, and efforts to further clarify physicians and patients.

Recent studies have shown conflicting results regarding the decision to use reference intervals (RI) of TSH suitable for the elderly or not [7,8]. On studies with review of medical records, it is difficult to use strict inclusion and exclusion criteria in the selection of subjects representing a control group. Several bias may occur due to registration errors, and omission of information that were not actively taken from the patient. The consequent inclusion of a significant number of subjects with potential thyroid or general disease or use of interfering medications as belonging to the control group that may alter the final data obtained is a real possibility. Thus, the theme is still far from being exhausted, and this topic is relevant regarding the issue of not to hyper diagnose subclinical hypothyroidism in the elderly who actually could have higher TSH, but at appropriate levels for their age.

Likewise, as FT4 is the test of choice for the confirmation of thyroid disease, it is also relevant to assess whether there is a need for RI specific for subjects above 60 years and 80 years or older.

The aim of this study was to test the hypothesis that it is necessary to use specific RI for TSH and FT4 for subjects over 60 years old, and over 80 years old, compare the values with those of young subjects, and analyze the impact of using specific TSH RI for these age groups in the screening of thyroid dysfunction.

## Materials and methods

Between March and December 2012, 1200 subjects of both sexes were evaluated prospectively distributed as follows: 120 males and 120 females in each of the following age ranges: 20–49, 50–59, 60–69, 70–79 and above 80 years, who attended a clinical laboratory to collect routine tests for which function evaluation and/or thyroid autoimmunity had not been requested. They were invited to participate in order to arrive at the laboratory on pre-determined weekdays, according to availability of the researchers. A questionnaire was applied by one of the researchers, through direct interviews with participating candidates, which consisted of: 1) Identification data, address and phone number of the participants, as well as the name and telephone number of their physician for communication in case of abnormal results; characterization of their color or race according to themselves; if they consider themselves as healthy individuals; personal or familial thyroid disease in the present or past; current medications (among these, medications containing iodine in the last six months), smoking, hospitalization due to illness or accident in the last six months (and what the disease was), and pregnancy (for females who had not had menopause). Subjects were from the metropolitan area of Rio de Janeiro, belonging to middle and upper social class, as declared income, 77% white, 18% mulatto and 5% black. Subjects with negative thyroid peroxidase antibodies (TPOAb) and thyroglobulin antibodies (TGAb), normal lipid profile, ultrasensitive C-reactive protein level (CRP), blood count, renal function, and absence of goiter on palpation were in the inclusion criteria. The measurement of urinary iodine was not done since the salt iodization in Brazil is determined by federal law [9] and the authors have shown in previous study that the intake iodine in the Rio de Janeiro population is sufficient [10]. In addition, the National Health Surveillance Agency (ANVISA), which is the regulatory agency under the Ministry of Health, conducted a review of salt iodization in the samples used in Rio de Janeiro in the year before beginning of the study confirming that it was appropriate [11].

Exclusion criteria included past or present history of thyroid disease, previous thyroid surgery, family history of thyroid disease, TSH <0.1 mU/L or > 10.0 mU/L, as these results indicate a high probability of thyroid dysfunction [12], palpable goiter, smoking habit, use of medicines known as possible analytical or physiological interference on measurement of TSH or FT4 in the past three months (medicines and contrasts containing iodine in the past six months) mentioned in the list of medications and other drugs that may interfere with TSH and/or FT4 measurements [13-24], antidepressants, hospitalization in the past six months and pregnant females. As changes in thyroid morphology are significantly higher among the elderly, we assessed whether this was a factor in generating different results in the selection of subjects for the study. A thyroid ultrasonography (US) was performed on 687 subjects with exclusion of 24.1% who presented some thyroid change. More than 120 subjects still remained in each group. Comparing TSH and FT4 values between subjects who had normal US and those who did not perform US, there was no statistically significant difference (data not shown). Therefore, as this exam was not essential in the selection of subjects as controls, all of the initially selected subjects could be enrolled in the study, except those who were initially excluded and had been replaced by the same number of other subjects of the same age and sex in order of maintaining the initial number to be studied.

### List of medications and other drugs that may interfere with TSH and/or FT4 measurements

2-3-dimercatopropanol, 2-4-dinitrophenol, 5-fluorouracil, 5-hydroxytryptophan

Acetazolamide, acetylsalicylic acid, alpha adrenergic blockers, aminoglutethimide, aminotriazole, amiodarone, androgens and other anabolic steroids, anphenona, anphetamines, antipyrine

Benserazide, beta adrenergic blockers, bexarotene, bromine, brompheniramine

Cadmium, carbamazepine, chromate, chromium picolinate, cimetidine, clofibrate, clomiphene, clomipramine, cobalt, complex anions, corticosteroids, cytostatics

Danazol, diphenylhydantoin, dinitrophenol, dobutamine, domperidone, dopamine and its agonists, other dopaminergic agents

Erythrosine, estrogens, ethionamide

Fenclofenac, fenoldopam, flunarizine, fluor, furosemide, fusaric acid

Growth hormone (GH), GH-Releasing hormone

Halofenate, heroin, heparin

Interleukins, iopanoic acid, other radiological contrasts, and other iodine-containing substances and drugs (potassium iodide and others), insulin-like Growth Factor-1, interferon

Ketoconazole

L-asparaginase, L-dopa inhibitors, levothyroxine, lithium, lovastatin,

Mefenamic acid, melatonin, metformin, methadone, methimazole, metoclopramide, mitotane

Nevirapine, niacin, nicotinic acid, nifedipine, NSAIDs, nitrate

O, p’-DDD, orphenadrine, opioids, oxcarbazepine

Para-aminobenzoic acid, perchlorate, perphenazine, phenidone, phenylbutazone, phenobarbital, pimozide, prazozin, primidone, propylthiouracil, pyridoxine

Quetiapine

Raloxifen, resorcinol, rifampicin, ritonavir, rubidium

Salsalate, serotonergics antagonists, somatostatin and its analogues, spironolactone, St. John’s Wort, stavudine, sulfonamides, sulfonylureas, sulpiride, steroids hormones

Tamoxifen, thiocyanate, thyroid hormones and their analogs, troleandomycin, tyrosine kinase inhibitors

Valproic acid

Drugs are listed in alphabetical order and not in possible importance as interfering in TSH or thyroid hormone values.

The study was approved by the Research Ethics Committee of the Hospital Clementino Fraga Filho, Universidade Federal do Rio de Janeiro (HUCFF/UFRJ) and individuals agreed to participate by signing the informed consent form.

### Collection of data and collection of samples

Serum was collected in the morning, in same species standard sampling tubes with separator gel. The measurements were done on the same day in primary tubes, after blood centrifugation at 3200 RPM for 15 min.

### Biochemical data

Serum TSH, FT4, TPOAb and TGAb were measured by electrochemiluminescence immunoassays on the Roche Modular Analytics® E170 (Roche Diagnostics Australia Pty Ltd, Castle Hill, NSW, Australia).

Serum TSH concentrations were measured by an immunometric method, with an intra-assay percentage coefficient of variation (% CV) of 3.0% at concentrations of 0.040 ± 0.001 mU/L, 2.7% at 0.092 ± 0.002 mU/L and 1.1% at 9.4 ± 0.1 mU/L. The reference interval provided by the manufacturer is 0.3- 4.2 mU/L. Serum FT4, TPOAb and TGAb concentrations were measured by competitive assays. For FT4, the % CV was 1.4% at FT4 concentrations of 0.7 ± 0.01 ng/dL, 1.8% at 1.3 ± 0.02 and 2.0% at 2.7 ± 0.1 ng/dL. The reference interval provided by the manufacturer is 0.9 -1.7 ng/dL. For TPOAb the intra-assay % CV is 6.3% at TPO concentrations of 21.3 ± 1.34 IU/mL, 5.1% at 51.2 ± 2.6 IU/mL and 2.7% at 473 ± 12.7 IU/mL. According to the manufacturer, individuals without thyroid disease score lower than 34 IU/mL. For TGAb the intra-assay % CV is 4.9% at TG concentrations of 47.2 ± 2.3 IU/mL, 1.3% at 588 ± 7.4, and 1.3 at 3289 ± 42.0 IU/mL TGAb concentrations. According to the manufacturer, individuals without thyroid disease score lower than <115 IU/mL.

### Statistical analysis

Data were analyzed using the program GraphPad Prism®, version 6.0 (GraphPad Software, Inc, California). In order to assess the Normal distribution of both data series (TSH and FT4) Kolmogorov-Smirnov tests were performed. Logarithmic 10 was used for analysis. TSH and FT4 were calculated for each subgroup and gender from 20 to 49 years, then, by 10-year age range until 80 years; all those aged older than 80 were grouped together. Descriptive analysis of serum TSH was reported as medians and 25% and 75% percentiles because it is not normally distributed. Two tailed Mann–Whitney test and Kruskal-Wallis test were used to compare the nonparametric TSH distributions in different subpopulations. Evaluation between two subgroups was made by independent test of Dunn multiple comparisons. Means and standard deviations were calculated for FT4, since it has showed a normal distribution. For comparing FT4 between all groups, two-way ANOVA tests were used when more than two data series were analyzed, and Student test in the case of two data series.

Outlying observations were calculated using the test proposed by Dixon. In all cases, the level of significance used was 0.05 [25]. For the RI, for both hormones 2.5% and 97.5% were taken. We used the method of Harris and Boyd [26] to decide whether it was necessary to separate the reference values for gender. According to this method, to calculate the statistical significance of the difference between the means of groups standard normal deviation (z), if the value z is less than 3 means there is no need to reference values separated by gender.

To calculate correlations between TSH and FT4 levels in all age groups, TSH data were transformed into logarithmic 10. With normal distribution the two-tailed Pearson test was used; it was considered statistically significant if a p-value was less than 0.05. Pearson test was also used to analyze, individually, correlations between TSH and age and FT4 and age.

## Results

### TSH data

**Figure 1:**
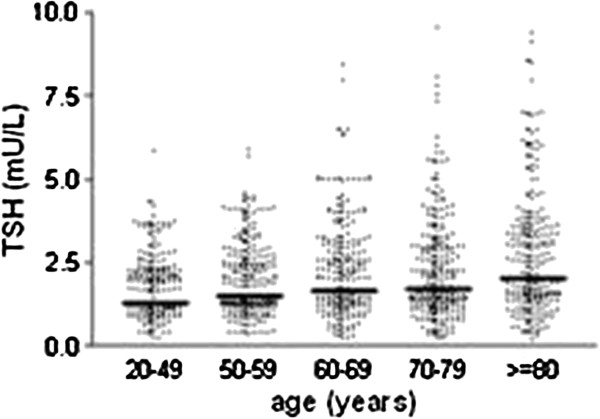
**Graph-distribution of TSH among different age groups.** The transverse line marks median values.

The reference group was comprised of 50% females and 50% males. The mean age by gender in each age subgroups are in Table 1. TSH data analysis by age or gender exhibited a non-Gaussian distribution. In each age group, there was no significant difference in serum TSH median between males and females (Table 1). Therefore, considering these factors, all individuals were eligible in each group for the establishment of reference intervals. Statistical parameters of TSH measurements are listed in Figure 1 and in Table 1. Analysis of median TSH as a whole group showed that there was a significant increase of this hormone with age (p < 0.001), but the analysis of independent subgroups, 20–49 years old *versus* 50–59 years old (p > 0.05), and 60–69 years old *versus* 70–79 years old (p > 0.05), showed no statistically significant difference. These data confirm that different RI for three major age groups should be used: 20 to 59 years, 60–79 years and 80 years or more. RI calculated for each sub-group are shown in Table 1.

**Table 1:** Statistical data of TSH measurements

	**Age groups**				
	**20-49**	**50-59**	**60-69**	**70-79**	**≥80**
Mean age females (years old)	34.3	54.1	64.2	74.4	84.4
Mean age males (years old)	35.2	53.9	64.2	74.2	86.7
Median TSH in females	1.5		1.7		2.0
Median TSH in males	1.5		1.8		2.1
z values and p values between gender	z = -1.45 p = 0.30	z = -0.60 p = 0.69	z = -0.43 p = 0.54	z = -0.78 p = 0.40	z = -0.14 p = 0.99
Minimum TSH	0.3	0.4	0.2	0.3	0.2
Maximum TSH	5.8	5.9	8.4	9.5	9.3
25% TSH percentile	1.1	1.2	1.7	1.7	2.0
75% TSH percentile	2.2	2.6	2.8	3.0	3.5
Lower 95% CI	1.6	1.8	2.0	2.1	2.5
Upper 95% CI	1.9	2.1	2.4	2.5	2.9
Median TSH assumed	1.5		1.7		2.0
Minimum and maximum TSH RI assumed	0.4 – 4.3		0.4 – 5.8		0.4 – 6.7

RI: reference interval SD: standard deviation TSH in mU/L.

### FT4 data

**Figure 2:**
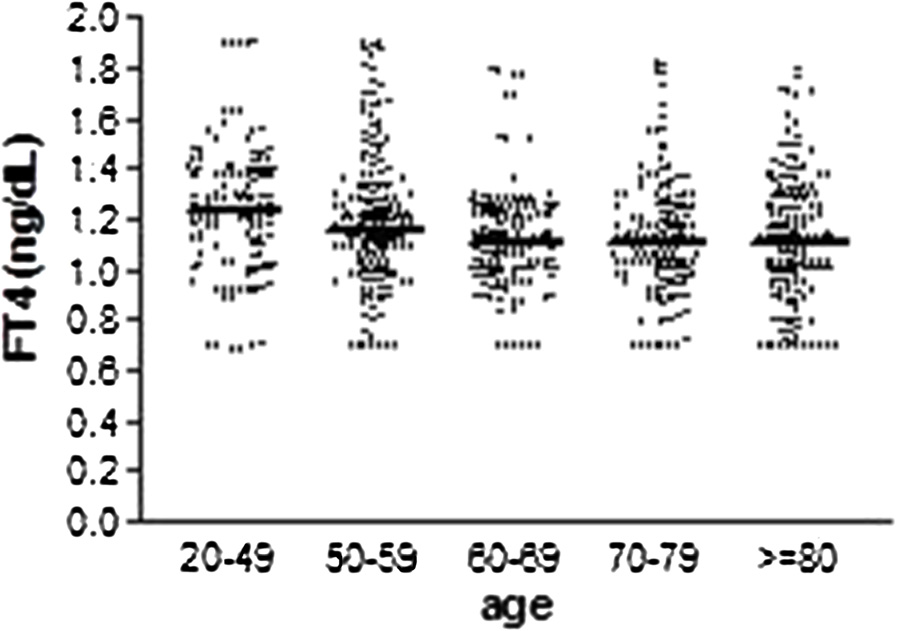
**Graph-distribution of FT4 among different age groups.** The transverse line marks median values.

FT4 exhibited a Gaussian distribution. Data of FT4 measurements are shown in Figure 2, and statistical parameters are listed in Table 2. Analysis of FT4 mean ± standard deviation (SD) shows that there is a significant reduction of the hormone with age as a whole group (p < 0.0001). However, despite a tendency to fall in FT4 with increasing age, the independent comparison test between the sub-groups showed that there was no statistically significant difference between those over 60 years old. Reference intervals calculated for FT4 are shown in Table 2.

**Table 2:** Statistical data of FT4 measurements

	**Age groups**				
	**20-49**	**50-59**	**60-69**	**70-79**	**≥80**
Mean FT4 ± SD in females	1.2 ± 0.03	1.2 ± 0.24	1.1 ± 0.22	1.2 ± 0.24	1.1 ± 0.24
Mean FT4 ± SD in males	1.3 ± 0.02	1.2 ± 0.25	1.1 ± 0.23	1.1 ± 0.22	1.1 ± 0.23
z values and p values between gender	z = 3.02 p = -0.0001	z = 1.26 p = 0.28	z = 0.70 p = 0.31	z = -1.63 p = 0.08	z = 0.39 p = 0.31
Minimum level FT4	0.7	0.7	0.7	0.7	0.7
Maximum level FT4	1.9	1.9	1.8	1.8	1.8
Lower FT4 95% CI	1.2	1.2	1.1	1.1	1.1
Upper FT4 95% CI	1.3	1.2	1.2	1.2	1.2
Mean FT4 ± SD assumed for both genders	1.2 ± 0.3	1.2 ± 0.3	1.1 ± 0.2	1.1 ± 0.2	1.1 ± 0.2
Minimum and maximum FT4 RI assumed for both genders	0.7 – 1.9		0.7 – 1.7		

RI: reference interval SD: standard deviation FT4 in ng/dL.

Regarding the correlation between TSH and FT4, a high level of significance was observed in all age sub-groups, independently analyzed (p < 0.0001), 20–49 years old: Pearson r = -0.4641, 95% confidence interval (CI) = -0.4961 to -0.2926, R squared = 0.1652; 50–59 years old: Pearson r = -0.3862, 95% CI = -0.4881 to -0.2739, R squared = 0.1492; 60–69 years old: Pearson r = -0.4653, 95% CI = -0.5583 to -0.3607, R squared = 0.2165; 70–79 years old: Pearson r = -0.4946, 95% CI = -0.5839 to -0.3934, R squared = 0.2446; 80 years old and over: Pearson r = -0.3951, 95% confidence interval = -0.4961 to -0.2835, R squared = 0.1561.

In order to assess whether the RI obtained in this study had clinical impact regarding the use of RI defined by the manufacturer in screening for thyroid disease, we compare the percentage of subjects who had TSH below or above the RI defined by age range obtained from this study with the values without segmentation by age. The results are showed in Table 3.

**Table 3:** **Comparison of data of TSH** (**mU**/**L**) **obtained regarding RI defined by the manufacturer** (**without segmentation by age**), **with the results of this study that redefined RI specific for age range**

	**Results of TSH below RI (mU/L)**			**Results of TSH upper RI (mU/L)**		
**Age ranges (y. old)**	**Defined by the manufacturer**	**With segmentation by age in this study**	Difference*	**Defined by the manufacturer**	**With segmentation by age in this study**	Difference*
**< 60**	0.4%	0.8%	+ 0.4%	1.7%	1.5%	- 0.2%
**60-79**	0.4%	2.1%	+ 1.7%	9.2%	2.7%	- 6.5%
**≥ 80**	0.4%	0.8%	+ 0.4%	14.5%	2.0%	- 12.5%

*Refers to percentage of subjects detected with TSH levels out of reference interval (*RI*) established by the manufacturer *versus* those where TSH was obtained in this study and *RI* was segmented by age.

## Discussion

Usually, for the interpretation of a laboratory test, clinical laboratories use RI provided by the manufacturer of lab kits. The result of a patient’s test is then compared to this RI in order to diagnose whether it is normal or not. TSH concentration is the most sensitive test to reliably detect thyroid function abnormalities and is used as the screening test for studying thyroid function because of the inverse log-linear relationship between circulating TSH and FT4 concentrations [27,28].

This study, conducted prospectively with a reference population, showed that TSH increases progressively and significantly with age. The value of z less than 3.0 in all age groups indicates that the same RI should be adopted for both genders. On the other hand, FT4 tends to decrease with age. In the age groups above 60 years, FT4 values are equal between males and females. In subjects younger than 60, the value of z pointed that FT4 is lower in males than in females. A study of Kratzsch et al. also reports lower FT4 in males than in females [29]. However, as in our study, z result (3.0249) is very close to the cutoff point we consider that in the clinical routine, it is appropriate to use the same RI to both genders, the same way as in older subjects.

The lower TSH limit defined in this study of 0.4 mU/L is the same for all age groups over 60 years old, and does not differ from the lower limit of younger people. This value is in accordance with previous study using third generation immunometric TSH methodology, that refers to the lower TSH reference limit as approximately 0.3 to 0.4 mU/L, irrespective of the population studied or the method used [30]. The value found in this study, very slightly higher than that reported by the manufacturer, has no impact on an eventual reclassification of how many people have low TSH, as the difference between one and other is under 2%.

Association between TSH and age is highly significant. The median is 1.5 mU/L in people under 60 years old, increases to 1.7 mU/L from 60 to 79 years old, and to 2.0 mU/L for those aged 80 or more. Upper TSH limit increases from 4.3 mU/L under 60 years old, to 5.8 mU/L in the range of 60 to 79 years old and to 6.7 mU/L in those very old subjects, over 80. So, although the manufacturer’s TSH kit is suitable for subjects less than 60 years of age, the same is not true for those 60 years or more, in which the limits are significantly higher.

Within the age ranges of 60–79 years and 80 years or over, a significant percentage of subjects are reclassified as having not elevated TSH if age-specific RI is adopted. The same does not occur in young subjects. According to the RI for each age group obtained in this study, 6.5% of subjects between 60 and 70 years and 12.5% of those with 80 years or more have less misdiagnosis of elevated TSH, leading to 19% reclassification from hypothyroidism to normal, using these criteria. With regard to the effects of age on serum TSH levels, other epidemiological studies indicate that the population’s mean TSH levels increase with age [4,31,32]. The NHANES III was the best designed in selecting a control population, and data obtained were similar to the current study, indicating that increase in TSH is probably a physiological event for the elderly [33,34] or that this increase may be due to the presence of TSH isoforms with low bioactivity [35]. In each scenario one can avoid excessive diagnosis of subclinical hypothyroidism in the elderly adopting specific RI. And, to clarify the second hypothesis, the answer would be the development and clinical use of technologies to quantify only isoforms with normal TSH bioactivity.

Failure to find these differences in RI between the young and the elderly reported in other studies may be due to retrospective studies based, or selection choosing populations to be reference for which strict selection criteria were not applied through specific questionnaires and appropriate physical examination to exclude factors such as thyroid dysfunction in the subject or in the family, interfering medications, other illnesses, recent hospitalizations, smoking habits and goiter.

Like TSH, FT4 has the same minimum reference value for all age groups. This value of 0.7 ng/dL however, is lower in relation to that defined by the manufacturer (0.9 ng/dL) in all groups. In relation to the maximum reference value, the level of 1.7 ng/dL is suitable to be used for subjects 60 years old and over. However, although this is not the scope of this study, the results obtained by us suggest that a higher reference superior value (1.9 ng/dL) should be used for young individuals.

Previous study demonstrated that FT4 remained relatively unchanged with age [36]. These data can be difficult to interpret because evaluation of thyroid function in the elderly is often complicated by the increased prevalence of chronic illness and the use of medication. In the present study, subjects with one or more of these factors were excluded. There is evidence that a low activity of thyroid hormone might be beneficial in the elderly. Low levels of FT4 have been associated with a better survival in elderly subjects [8,33,37,38]. On the other hand, even thyroid hormone levels within the normal range might be associated with thyroid hormone-related endpoints. As an example, in euthyroid subjects, especially the elderly, FT4, regardless of TSH levels is associated with atrial fibrillation, and lower physical performance [39,40]. One hypothesis is that these lower levels of thyroid hormone could possibly serve as an adaptive mechanism to prevent catabolism in the elderly [41].

We consider relevant the data obtained in this age-related prospective study, once it is very important to distinguish between normal and mildly elevated serum TSH concentrations in elderly subjects. Elevations of TSH in young individuals even light and without decreasing FT4 characterize subclinical hypothyroidism or minimal thyroid dysfunction and are related to comorbidities such as dyslipidemia, adverse obstetric events, impact on cognition, quality of life, cardiovascular events, and evolution to clinical hypothyroidism [42]. These effects have no correspondence in the elderly, since there is no evidence of these effects in this age group [43,44]. There is a consensus that subjects with TSH concentrations above 10.0 mU/L should be treated. However, according to Garber et al., in the Clinical Practice Guidelines for Hypothyroidism, very mild TSH elevations in older individuals, under this level, may not reflect subclinical thyroid dysfunction, but rather be a normal manifestation of aging. While the normal TSH reference may need to be narrowed range for some subpopulations [27,45], the normal RI may widen with aging [24]. This confirms that not all patients who have mild TSH elevations are hypothyroid and therefore would not require thyroid hormone therapy. These data are also relevant for the monitoring of subjects with hypothyroidism, since the target for TSH in levothyroxine treated subjects should be higher in elderly people. Pitfalls in the interpretation of TSH were carefully excluded in this study. Abnormal levels are observed in various non-thyroidal diseases and other conditions, but the effect of possible changes secondary to thyroid diseases were preventable excluding subjects who had used medication containing iodine during the previous 6 months, had had hospitalization, smokers, as well as those not using any medication that may alter minimally TSH or thyroid hormones.

Concluding, this data shows that prevalence of subclinical hypothyroidism is overestimated in the elderly, in almost 20% of subjects, unless age-specific RI is used. This might improve diagnostic accuracy and reduce the need of confirmatory unnecessary tests.

## Competing interests

The author’s declare that they have no competing interests.

## Authors’ contributions

RF did the study design, conducted the field study, made the final selection of study participants; computed the data and wrote the text. CRC made the initial guidance on the statistical requirements for the study, led and oversaw all statistical work. FA did the statistical field study under the supervision of CRC, being assisted during the course of the study also by the statistical mentioned in the acknowledgments. MV was the general supervisor of the study. He had discussions with the staff and the necessary modifications to the original design of the study; he revised, and made necessary adaptions to the final text and made the final approval of the text submitted to the journal. All authors read and approved the final manuscript.

## References

[B1] United Nations publication The Sex and Age distribution of the world populations Sex and Age Popul Div, United Nations Secretariat, the 1998 Revis 1998 6 2 6 No. E.99.XIII.8

[B2] Chiovato L Mariotti S Pinchera A Thyroid diseases in the elderly Baillieres Clin Endocrinol Metab 1997 6 251 270 10.1016/S0950-351X(97)80272-8 9403122

[B3] Canaris GJ Manowitz NR Mayor G Ridgway EC The Colorado thyroid disease prevalence study Arch Intern Med 2000 6 526 534 10.1001/archinte.160.4.526 10695693

[B4] Hollowell JG Staehling NW Flanders WD Hannon WH Gunter EW Spencer CA Braverman LE Serum TSH, T_4_, and Thyroid Antibodies in the United States Population (1988 to 1994): National Health and Nutrition Examination Survey (NHANES III) J Clin Endocrinol Metab 2002 6 489 499 10.1210/jc.87.2.489 11836274

[B5] Ceriotti F Prerequisites for use of common reference intervals Clin Biochem Rev 2007 6 115 121 17909616 PMC1994109

[B6] Horowitz GL Altaie S Boyd JC Ceriotti F Garg U Horn P Pesce A Sine HE Zakovski J Defining, Establishing, and Verifying Reference Intervals in the Clinical Laboratory; Approved Guideline – Third Edition Clin Lab Stand Inst 2008 6 C28 A3

[B7] Kahapola-Arachchige KM Hadlow N Wardrop R Lim EM Walsh JP Age-specific TSH reference ranges have minimal impact on the diagnosis of thyroid dysfunction Clin Endocrinol 2012 6 773 779 10.1111/j.1365-2265.2012.04463.x 22703566

[B8] Vadiveloo T Donnan PT Murphy MJ Leese GP Age- and gender-specific TSH reference intervals in People with no obvious Thyroid disease in Tayside, Scotland: the Thyroid Epidemiology, Audit, and Research Study (TEARS) J Clin Endocrinol Metab 2013 6 1147 1153 10.1210/jc.2012-3191 23345094

[B9] Lei 1944/53 | Lei n° 1.944, de 14 de agosto de 1953 (Law 1944/53 | Law No. 1,944, of August 14, 1953) [http://presrepublica.jusbrasil.com.br/legislacao/128787/lei-1944-53]

[B10] Netto LS Coeli CM Micmacher E Mamede SC Nazar LO Correa EK Arrastia M Galvão D Buescu A Vaisman M Longitudinal study of pituitary-thyroid axis in pregnancy Arq Bras Endocrinol Metab 2004 6 493 498 10.1590/s0004-27302004000400009 15761512

[B11] Resultado do Monitoramento do Teor de Iodo no Sal Results of monitoring of iodine in salt 2011 [http://portal.anvisa.gov.br/wps/wcm/connect/7d7bb4804be9dbf18c50ddbc0f9d5b29/Relat%C3%B3rio+Pro.Iodo+2011.pdf?MOD=AJPERES]

[B12] Surks MI Ortiz E Daniels GH Sawin CT Col NF Cobin RH Franklin JA Hershman JM Burman KD Denke MA Gorman C Cooper RS Weissman NJ Subclinical thyroid disease: scientific review and guidelines for diagnosis and management JAMA 2004 6 228 238 10.1001/jama.291.2.228 14722150

[B13] Oppenheimer JH Schwartz HL Dillman W Surks MI Effect of thyroid hormone analogues on the displacement of ^125^I-L-triiodothyronine from hepatic and heart nuclei in vivo: possible relationship to hormonal activity Bioch Bioph Res Commun 1973 6 544 550 10.1016/0006-291X(73)91177-7 4357424

[B14] Liewendahl K Majuri H Helenius T Thyroid function tests in patients on long-term treatment with various anticonvulsivant drugs Clin Endocrinol 1978 6 185 191 10.1111/j.1365-2265.1978.tb01493.x 346267

[B15] Smith PJ Surks MI Multiple effects of 5,5’-diphenylhydantoin on the thyroid hormone system Endocr Rev 1984 6 514 524 10.1210/edrv-5-4-514 6094173

[B16] Spencer C Eigen A Shen D Duda M Qualls S Weiss S Nicoloff J Specificity of sensitive assays of thyrotropin (TSH) used to screen for thyroid disease in hospitalized patients Clin Chem 1987 6 1391 1396 3301067

[B17] Larkin JG Macphee GJA Beastall GH Brodie MJ Thyroid hormone concentrations in epileptic patients Eur J Clin Pharmacol 1989 6 213 216 10.1007/BF00558149 2501100

[B18] Davies PH Franklyn JA The effects of drugs on tests of thyroid function Eur J Clin Pharmacol 1991 6 439 451 10.1007/BF00315221 1884719

[B19] Stockigt JR ree thyroid hormone measurement A Crit appraisal 2001 Endocrinol Metab Clin North Am 2001 6 265 289 10.1016/s0889-8529(05)70187-0 11444163

[B20] Steele BW Wang E Klee GG Thienpont LM Soldin SJ Sokoll LJ Winter WE Fuhrman SA Elin RJ Analytic bias of thyroid function tests: analysis of a college of American Pathologists fresh frozen serum pool by 3900 clinical laboratories Arch Pathol Lab Med 2005 6 310 317 15737023 10.5858/2005-129-310-ABOTFT

[B21] Crofton KM Thyroid disrupting chemicals: mechanisms and mixtures Int J Androl 2008 6 209 223 10.1111/j.1365-2605.2007.00857.x 18217984

[B22] Stockigt JR Lim CF Medications that distort in vitro tests of thyroid function, with particular reference to estimates of serum free thyroxine Best Pract Clin Endocrinol Metab 2009 6 753 767 10.1016/j.beem.2009.06.004 19942151

[B23] Beckett GJ Mechanisms behind the non-thyroidal illness syndrome: an update J Endocrinol 2010 6 1 13 10.1677/JOE-09-0412 20016054

[B24] Garber JR Cobin RH Gharib H Hennessey JV Klein I Mechanick JI Pessah-Pollack R Singer PA Woeber KA Clinical practice guidelines for hypothyroidism in adults: Cosponsored by the American Association of Clinical Endocrinologists and the American Thyroid Association Thyroid 2012 6 1200 1235 10.1089/thy.2012.0205 22954017

[B25] Dixon WJ Processing data for outliers Biometrics 1953 6 74 89 10.2307/3001634

[B26] Harris EK Boyd JC On dividing reference data into subgroups to produce reference ranges Clin Chem 1990 6 265 270 2302771

[B27] Baloch Z Carayon P Conte-Devolx B Demers LM Feldt-Rasmussen U Henry JF LiVosli VA Niccoli-Sire P John R Ruf J Smyth PP Spencer CA Stockigt JR Laboratory medicine practice guidelines. Laboratory support for the diagnosis and monitoring of thyroid disease Thyroid 2003 6 3 126 12625976 10.1089/105072503321086962

[B28] Benhaldi N Fliers E Visser TJ Reitsma JB Wiersinga WM Pilot study on the assessment of the setpoint on the hypothalamus-pituitary-tryroid axis in healthy volunteers Eur J Endocrinol 2010 6 323 329 10.1530/EJE-09-0655 19926783

[B29] Kratzsch J Fiedler GM Leichtle A Brügel M Buchbinder S Otto L Sabri O Matthes G Thiery J New reference intervals for thyrotropin and thyroid hormones based on national academy of clinical biochemistry criteria and regular ultrasonography of the thyroid Clin Chem 2005 6 1480 1486 10.1373/clinchem.2004.047399 15961550

[B30] D’Herbomez M Jarrige V Darte C Reference intervals for serum thyrotropin (TSH) and free thyroxine (FT4) in adults using the Access Immunoassay System Clin Chem Lab Med 2005 6 102 105 15653452 10.1515/CCLM.2005.017

[B31] Brochmann H Bjøro T Gaarder PI Hanson F Frey HM Prevalence of thyroid dysfunction in elderly subjects. A randomized study in a Norwegian rural community (Naerøy). Acta Endocrinol (Copenh) 1988 6 7 12 3381628 10.1530/acta.0.1170007

[B32] Boucai L Hollowell JG Surks MI An approach for development of age-, gender-, and ethnicity-specific thyrotropin reference limits Thyroid 2011 6 5 11 10.1089/thy.2010.0092 21058882 PMC3012447

[B33] Atzmon G Barzilai N Hollowell JG Surks MI Gabriely I Extreme longevity is associated with increased serum thyrotropin J Clin Endocrinol Metab 2009 6 1251 1254 10.1210/jc.2008-2325 19158193 PMC2682478

[B34] Surks MI Boucai L Age- and race- based serum thyrotropin reference limits J Clin Endocrinol Metab 2010 6 496 502 10.1210/jc.2009-1845 19965925

[B35] Estrada JM Soldin D Buckey TM Burman KD Soldin OP Thyrotropin isoforms – implications for TSH analysis and clinical practice Thyroid 2013 in press 10.1089/thy.2013.0119 PMC3949435 24073798

[B36] Mariotti S Franceschi C Cossarizza A Pinchera A The aging thyroid Endocr Rev 1995 6 686 715 8747831 10.1210/edrv-16-6-686

[B37] Gussekloo J van Exel E de Craen AJ Meinders AE Frölich M Westendorp RG Thyroid status, disability and cognitive function, and survival in old age JAMA 2004 6 2591 2599 10.1001/jama.292.21.2591 15572717

[B38] Van Den Beld AW Visser TJ Feelders RA Grobbee DE Lamberts SWJ Thyroid hormone concentrations, disease, physical function, and mortality in elderly Men J Clin Endocrinol Metab 2005 6 6403 6409 10.1210/jc.2005-0872 16174720

[B39] Gammage MD Parle JV Holder RL Roberts LM Hobbs FD Wilson S Sheppard MC Franklyn JA Association between serum free thyroxine concentration and atrial fibrillation Arch Intern Med 2007 6 928 934 10.1001/archinte.167.9.928 17502534

[B40] Heeringa J Hoogendoorn EH von der Deure WM Hofman A Peeters RP Hop WCJ den Heijer M Visser TJ Witterman JCM High-normal thyroid function and risk of atrial fibrillation. The rotterdam study Arch intern Med 2008 6 2219 2224 10.1001/archinte.168.20.2219 19001198

[B41] Peeters RP Thyroid function and longevity: New insights into an Old dilemma J Clin Endocrinol Metab 2009 6 4658 4660 10.1210/jc.2009-2198 19959751

[B42] Biondi B Cooper DS The clinical significance of subclinical thyroid dysfunction Endocr Rev 2008 6 76 131 17991805 10.1210/er.2006-0043

[B43] Laurberg P Andersen S Carle A Karmisholt J Knudsen N Pedersen IB The TSH upper reference limit: where are we at? Nat Rev Endocrinol 2011 6 232 239 10.1038/nrendo.2011.13 21301488

[B44] Tseng F-Y Lin W-Y Lin C-C Lee L-T Li T-C Sung P-K Huang K-C Subclinical hypothyroidism is associated with increased risk for All-cause and cardiovascular mortality in adults J Am col Cardiol 2012 6 730 737 10.1016/j.jacc.2012.03.047 22726629

[B45] Wartofsky L Dickey RA The evidence for a narrower thyrotropin reference range is compelling 2005 J Clin Endocrinol Metab 2005 6 5483 5488 10.1210/jc.2005-0455 16148345

